# *Cauliflower mosaic virus* Protein P6 Inhibits Signaling Responses to Salicylic Acid and Regulates Innate Immunity

**DOI:** 10.1371/journal.pone.0047535

**Published:** 2012-10-11

**Authors:** Andrew J. Love, Chiara Geri, Janet Laird, Craig Carr, Byung-Wook Yun, Gary J. Loake, Yasuomi Tada, Ari Sadanandom, Joel J. Milner

**Affiliations:** 1 Plant Science Research Theme, School of Life Sciences and Institute of Molecular, Cellular and Systems Biology, College of Medical, Veterinary and Life Sciences, University of Glasgow, Glasgow, Scotland, United Kingdom; 2 Istituto di Biologia e Biotechnologia Agraria, Consiglio Nazionale Delle Richerche, Pisa, Italy; 3 Institute of Molecular Plant Sciences, University of Edinburgh, King's Buildings, Edinburgh, United Kingdom; 4 Department of Biology, Duke University, Durham, North Carolina, United States of America; Friedrich-Alexander-University Erlangen-Nurenberg, Germany

## Abstract

*Cauliflower mosaic virus* (CaMV) encodes a multifunctional protein P6 that is required for translation of the 35S RNA and also acts as a suppressor of RNA silencing. Here we demonstrate that P6 additionally acts as a pathogenicity effector of an unique and novel type, modifying NPR1 (a key regulator of salicylic acid (SA)- and jasmonic acid (JA)-dependent signaling) and inhibiting SA-dependent defence responses We find that that transgene-mediated expression of P6 in Arabidopsis and transient expression in *Nicotiana benthamiana* has profound effects on defence signaling, suppressing expression of representative SA-responsive genes and increasing expression of representative JA-responsive genes. Relative to wild-type Arabidopsis P6-expressing transgenics had greatly reduced expression of *PR-1* following SA-treatment, infection by CaMV or inoculation with an avirulent bacterial pathogen *Pseudomonas syringae* pv tomato (*Ps*t). Similarly transient expression in *Nicotiana benthamiana* of P6 (including a mutant form defective in translational transactivation activity) suppressed *PR-1a* transcript accumulation in response to Agrobacterium infiltration and following SA-treatment. As well as suppressing the expression of representative SA-regulated genes, P6-transgenic Arabidopsis showed greatly enhanced susceptibility to both virulent and avirulent *P*st (titres elevated 10 to 30-fold compared to non-transgenic controls) but reduced susceptibility to the necrotrophic fungus *Botrytis cinerea*. Necrosis following SA-treatment or inoculation with avirulent *P*st was reduced and delayed in P6-transgenics. NPR1 an important regulator of SA/JA crosstalk, was more highly expressed in the presence of P6 and introduction of the P6 transgene into a transgenic line expressing an NPR1:GFP fusion resulted in greatly increased fluorescence in nuclei even in the absence of SA. Thus in the presence of P6 an inactive form of NPR1 is mislocalized in the nucleus even in uninduced plants. These results demonstrate that P6 is a new type of pathogenicity effector protein that enhances susceptibility to biotrophic pathogens by suppressing SA- but enhancing JA-signaling responses.

## Introduction

It is a paradigm that any micro-organism that functions as an effective pathogen must possess mechanisms either to suppress or evade the armoury of host defence responses. This is particularly true for biotrophic plant pathogens because they can only maintain a successful infection in the presence of living host cells. Plant pathogenic bacteria introduce dozens of pathogen-encoded effectors into the host via the Type III secretion system (TTSS) [1] and biotrophic fungal or oomycete pathogens may deliver hundreds of effectors across the haustorial membrane [2], [3] that suppress or modify plant defence responses.

Plant viruses are amongst the least genetically complex pathogens but must nevertheless maintain the ability to overcome host-defences. Much recent attention has focused on the role of RNA-silencing in anti-virus defence and the majority of plant viruses encode silencing suppressor proteins (VSSPs) [4]. As well as RNA-silencing, many plant viruses stimulate basal defence responses in compatible hosts; these include the global activation of Salicylic Acid (SA)-responsive and changes in the abundance of Jasmonic Acid (JA)-responsive genes [5], [6]. Exogenous application of SA or analogues can reduce accumulation or long-distance movement of a number of viruses, e.g. *Tobacco mosaic virus* (TMV) and *Cucumber mosaic virus* (CMV [7]–[9] and long distance movement of *Cauliflower mosaic virus* (CaMV) is inhibited in Arabidopsis (*Arabidopsis thaliana*) mutants in which SA-responsive defence pathways are constitutively activated [10]. CMV protein 2b, a VSSP, has also been implicated as modifying both JA- and SA-responses implying a possible link between silencing, SA and JA [6], [11], although in Arabidopsis SA-mediated antiviral defence against TMV and CMV appears to act partially independently of DICER activity [12]. These results demonstrate the importance of SA and JA signaling in defence responses following infection by either compatible or incompatible viruses.

CaMV is a plant pararetrovirus with an 8.0 kbp DNA genome and a worldwide distribution [13]. Hosts include members of the *Cruciferae* [13] and infections of Arabidopsis (*Arabidopsis thaliana*) make an excellent model pathosystem with which to study signaling and defence responses during compatible host-virus interactions. In Arabidopsis, CaMV-infection strongly stimulates activation of genes responsive to ethylene, reactive oxygen species and SA [14]. Up-regulation of markers such as *PR-1*, *BGL2* and *PR-5* is dependent on functional SA-signaling pathways. However, although Arabidopsis mutants in which SA-responsive defence pathways are constitutively activated show enhanced resistance to CaMV, genotypes that cannot accumulate SA e.g. *NahG* and *sid2-2* do not show any corresponding enhanced susceptibility [10]. Therefore, in wild-type Arabidopsis plants (but not in mutants in which Systemic Acquired Resistance (SAR) is constitutively activated) SA-dependent defence responses triggered by CaMV-infection may be ineffective in restricting virus multiplication and spread.

Measured on a whole plant basis, SA-responsive transcripts increase in abundance over time following CaMV-inoculation but leaf-by-leaf the situation is more complex. In individual leaves at the very early stages of virus invasion, levels of *PR-1* transcripts are high but they decrease as virus titres within the leaf increase [15], [16]. A plausible explanation for this strong inverse correlation between virus titre and *PR-1* expression in individual leaves, and for the lack of enhanced susceptibility in SA-deficient genotypes [10], is that a virus-encoded protein may be suppressing SA-signaling in infected cells.

CaMV gene VI encodes P6, a 62 kD multifunctional nuclear-cytoplasmic shuttle protein [17] with an essential role in virus replication [13]. P6 acts as a translational transactivator (TAV) promoting translation of downstream open reading frames (ORFs) on the polycistronic 35 S mRNA through a non-canonical mechanism [13], [18]. It also associates with actin filaments and plays a role in virus cell-to-cell movement [19], [20]. Gene VI is also the major genetic determinant of pathogenicity [21]–[23] and in some hosts P6 functions as an avirulence determinant triggering a hypersensitive response (HR) [24], [25]. Consistent with a role as a pathogenicity determinant, P6 can also act as a suppressor of RNA-silencing, interfering with DICER activity through an interaction with nuclear protein DRB4 [26], [27]. Here we have investigated whether P6 might play yet another role by suppressing SA-dependent defence responses.

We have previously reported that expression of P6 from a transgene in Arabidopsis results in a symptom-like phenotype and modifies ethylene and Auxin signaling responses [28]–[30]. We now show that in transgenic Arabidopsis and when transiently expressed in *Nicotiana benthamiana*, P6 dramatically decreases the abundance of representative SA-responsive transcripts and increases the abundance of JA-responsive transcripts. P6 expression results in increased levels and altered subcellular localization of NONEXPRESSOROFPATHOGENESISRELATED1 (NPR1), a key regulatory protein that controls many aspects of SAR in plants. We also show that in P6-expressing transgenic plants, susceptibility to virulent and avirulent strains of *Pseudomonas syringae pv* tomato (*Ps*t) is greatly enhanced, but susceptibility to the necrotrophic fungal pathogen *Botrytis cinerea* is greatly reduced. We conclude that P6 is a novel and unique pathogenicity effector that specifically targets basal defence by profoundly altering signaling responses to SA and JA.

## Results

### Expression of CaMV-P6 from a transgene in Arabidopsis and transiently in *N. benthamiana* suppresses transcriptional up-regulation of SA-dependent marker genes in response to infection

**Figure 1:**
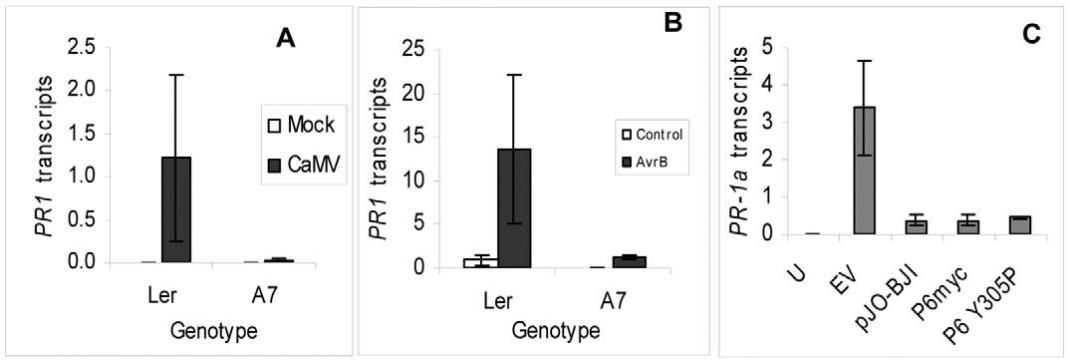
Quantification of *AtPR-1* and *NbPR-1a* transcripts in response to pathogens. (A) *PR-1* transcripts in mock-inoculated and CaMV-infected NT (L*er*) and P6-transgenic (A7) Arabidopsis, determined by qPCR. Bars show mean levels (in arbitrary units) of 3 independent biological samples each comprising pooled tissue from 3 plants. Samples were harvested 14 dpi. Error bars show standard deviations. (B) *PR-1* transcripts in uninoculated controls and *Ps*t (*AvrB*) inoculated NT (L*er*) and P6-transgenic (A7) Arabidopsis, determined by qPCR. Bars show mean levels (in arbitrary units) of 3 independent biological samples each comprising pooled inoculated leaves from 3 plants. Samples were harvested 48 h after infiltration. Error bars show standard deviations. (C) *PR-1a* transcripts, determined by qPCR, in *N. benthamiana* leaves harvested 48 h after agroinfiltration. Samples were (U) uninfiltrated leaves, and leaves infiltrated with Agrobacterium carrying the following vectors (EV) pJO530, (pJO-BJI) pJO-BJI, (P6myc) pGWB-P6myc, (P6Y305P) pGWB-P6Y305P. Bars show mean levels (in arbitrary units) of 3 independent biological samples each comprising 3 pooled infiltrated leaf sections. Error bars show standard deviations.

To test if P6 might be interfering with SA-signaling during CaMV infection we inoculated transgenic Arabidopsis plants which constitutively expresses high levels of P6 [28] with CaMV, and compared the expression of *PR-1* (a reliable marker for SA-dependent defence) [10], [31] with CaMV-infected non-transgenic (NT) plants in the same ecotypic (L*er*) background. Transcripts were measured by Real Time qPCR in samples harvested at 14 dpi a time at which we have previously showed that SA-responsive markers are strongly up-regulated in CaMV-infected wild-type plants [14]. In a typical P6-transgenic line (A7) virus levels at 14 dpi (measured by q-PCR) were 26±4% those in NT; we have previously reported similarly reduced virus titres in P6-transgenic lines [26]. In NT plants, CaMV-infection stimulated the expected large increase in abundance of *PR-1* transcripts. In contrast, *PR-1* transcripts accumulated in A7 to levels that were only 2.6±0.3% the levels in infected NT and only slightly greater than in uninfected NT plants (figure 1A). Although virus titres in the P6-transgenic plants were lower than in NT, as we have reported previously levels of *PR-1* transcripts are not directly proportional to virus titres but rise sharply around 8 dpi (at which time virus levels are still low) [14], [15]; we therefore think it unlikely that the large reduction in *PR-1* transcript levels in the P6-transgenic relative to NT lines is attributable to the much more modest relative reduction in virus titres.

To determine whether the effect was specific to CaMV-infection, we quantified *PR-1* transcripts 48 h after inoculation with *Pseudomonas syringae pv* tomato (*Ps*t) (*AvrB*), which is avirulent on L*er* and triggers a strong gene-for-gene response [32]. Transcript levels in *Ps*t (*AvrB*)-inoculated leaves of NT plants showed the anticipated large increase compared to uninoculated controls (figure 1B). In leaves of P6-transgenic plants inoculated with *P*st (*AvrB*), *PR-1* transcripts only accumulated to levels that were approximately one tenth those in *P*st (*AvrB*)-inoculated NT. Thus, expression of P6 from a transgene inhibited the expression of a SA-dependent marker gene both in response to a virus-infection and in a gene-for-gene response to a bacterial infection.

To assess if this novel function was limited to Arabidopsis or could be manifested in other plant species we took advantage of the ability of Agrobacterium (*Agrobacterium tumefaciens*) to trigger Pathogen-Associated Molecular Pattern (PAMP)-driven defence responses when infiltrated into *Nicotiana* leaves [33]. We transiently expressed P6 in *Nicotiana benthamiana* by infiltrating leaves with Agrobacterium carrying an appropriate Ti binary expression vector and measured the expression of *NbPR-1a* a homologue of the SA-responsive marker genes *NtPR-1a* in *N. tabacum* and *AtPR-1* in Arabidopsis [34]. Transcript levels were measured in leaves agroinfiltrated with Ti binary plasmid pJO-BJI [28] in which expression of P6 is driven from a *35S* promoter; controls comprised leaves agroinfiltrated with the empty parent vector pJO530 (EV). The expression of P6 in agroinfiltrated leaves was confirmed in western blots using anti-P6 antibody; these gave a strong immunoreactive band of the expected size in leaves infiltrated with pJO-BJI, but not in uninfiltrated controls or leaves agroinfiltrated with EV (supplementary figure S1A). Levels of P6 in *N. benthamiana* leaves were slightly higher than in the P6-overexpressing transgenic Arabidopsis line A7; we have previously shown these to be similar to levels in CaMV-infected Arabidopsis [28].

Agroinfiltration with EV stimulated an increase in *NbPR-1a* transcripts of more than three orders of magnitude compared to uninfiltrated controls, reflecting the PAMP-driven engagement of basal defence [33]. In leaves agroinfiltrated with pJO-BJI (for transient expression of P6) the *NbPR-1a* transcripts were more abundant than in the untreated control, but were however consistently ∼10-fold less abundant than in EV-agroinfiltrated leaves (figure 1C). These results taken together indicate that P6 is able to suppress representative SA-dependent responses to different pathogens in two unrelated plant species, Arabidopsis and *N. benthamiana*.

P6 plays an essential role in CaMV-replication facilitating the translation of downstream ORFs on the polycistronic 35S RNA by translational transactivation (TAV). To determine whether the TAV activity of P6 was required for suppressing PAMP-responsive *NbPR-1a* expression, we agroinfiltrated *N. benthamiana* with a construct expressing a mutant form P6^Y305P^, in which the conserved Tyrosine at amino acid 305 has been substituted for a Proline, a change that abolishes TAV activity [35]. Since P6^Y305P^ was constructed in a different binary vector pGWB17 [36], we used as an additional control a third construct P6myc that expresses wild-type P6 (with a C-terminal myc tag) from the same pGWB17 vector. P6 protein levels in P6myc and P6^Y305P^-infiltrated leaves were much lower than in leaves agroinfiltrated with pJO-BJI and although P6myc and P6^Y305P^ proteins were readily detectable in western Blots using an anti-myc antibody they gave only faint bands using anti-P6 antibodies making it difficult to accurately compare expression of the three constructs in the same blot. To better compare expression from the three constructs we quantified P6 transcripts by qPCR (Supplementary figure S1B) Transcript levels from P6myc and P6^Y305P^ were 7% and 15% respectively those from pJO-BJI but despite the much lower levels of expression of P6^Y305P^ and P6myc, *NbPR-1a* transcript levels were essentially identical to those with pJO-BJI and ∼10% the level in plants agroinfiltrated with EV. Therefore the ability of P6 to suppress expression of a SA-responsive marker gene does not require functional TAV activity.

### P6 reduces levels of SA-responsive gene transcripts in SA-treated and in untreated plants

**Figure 2:**
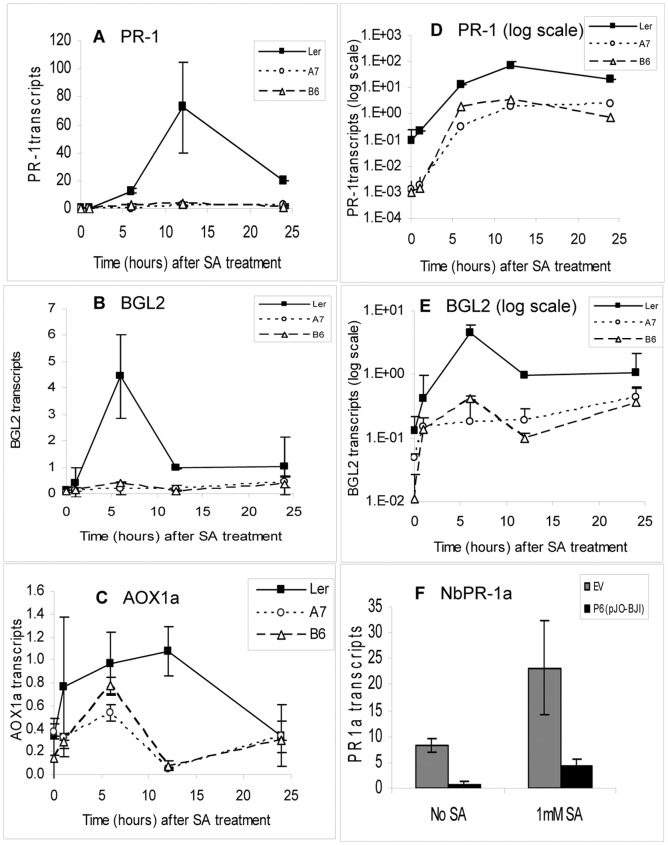
Quantification of transcripts of SA-responsive genes in P6-expressing and non-expressing plants following SA-treatment. (A) *PR-1a* transcripts (in arbitrary units), determined by qPCR, in P6-transgenic (A7 and B6) and NT (L*er*) Arabidopsis following treatment with 1.0 mM SA. Each point represents mean levels of 3 independent biological samples each comprising pooled tissue from 3 plants harvested at intervals from 0 to 24 h. Error bars show standard deviations. (B) *BGL2* transcripts (in arbitrary units), determined by qPCR, in P6-transgenic (A7 and B6) and non transgenic (L*er*) Arabidopsis following treatment with 1.0 mM SA as in figure 2A above. (C) *AOX1a* transcripts (in arbitrary units), determined by qPCR, in P6-transgenic (A7 and B6) and non transgenic (L*er*) Arabidopsis following treatment with 1.0 mM SA as in figure 2A above. (D) Data presented in (A) but with abundance of *PR-1a* transcripts plotted on a logarithmic scale. Error bars (positive only) show standard deviations. (E) Data presented in (B) but with abundance of *BGL2* transcripts plotted on a logarithmic scale. Error bars (positive only) show standard deviations. (F) *PR-1a* transcripts, determined by qPCR, in *N. benthamiana* leaves following treatment with 1.0 mM SA. Leaves were infiltrated with Agrobacterium carrying either empty vector (pJO530) or a binary vector expressing P6 (pJO-BJI) as in figure 1C above. After 48 h leaves were sprayed with 1 mM SA; controls were left untreated. Tissue was harvested 12 h later and PR-1a transcripts were quantitated by qPCR. Bars show mean levels (in arbitrary units) of 3 independent biological samples each comprising 3 pooled infiltrated leaf sections. Error bars show standard deviations.

The very low levels of *AtPR-1* and *NbPR-1a* transcripts in plants exposed to pathogens suggested that P6 was most likely interfering with SA-signal transduction. We therefore tested the responsiveness of P6-transgenic plants to exogenous application of SA. NT (L*er*) and two independent P6-transgenic lines, A7 and B6 were sprayed with 1.0 mM SA. We then quantified transcripts of three representative SA-responsive marker genes *PR-1*, *BGL2* and *AOX1A* at intervals up to 24 h after treatment (figure 2A, B and C). SA-treatment of NT elicited the expected strong time-dependent increase in *PR-1* transcripts (up to a maximum of ∼700-fold) and a slightly smaller but somewhat more rapid increase in *BGL2* transcripts. In contrast, in P6-transgenics levels of both transcripts remained very low following application of SA. SA stimulated a more modest increase (up to 4-fold) in *AOX1A* transcript levels in NT; again in P6-transgenics levels increased only slightly following application of SA. To take advantage of the sensitivity of qPCR, which allows accurate quantification of transcript levels over a range of several orders of magnitude, and to allow accurate comparison of transcript levels in untreated as well as SA-treated plants we plotted the data on a log scale (figure 2D and E). Levels of *BGL2* and *PR-1* transcripts in untreated P6 transgenic lines were about 1 to 2 logs lower than in untreated NT plants. Following SA treatment transcript levels showed similar time-dependent increases *relative to untreated plants* in all three backgrounds but at each of the individual time point, absolute levels in the P6-transgenic lines were always 1 to 2 logs lower than in NT plants. For *AOX1a* transcripts, differences in levels between P6-transgenic and NT plants were much smaller (figure 2C) and comparison on a log scale was not informative.

To establish whether a similar effect was occurring in *N. benthamiana*, leaves were agroinfiltrated with either pJO-BJI or EV. After 48 h (to allow expression of P6) they were sprayed with SA and *NbPR-1a* transcripts were quantified 12 h later (figure 2F). In agroinfiltrated plants not treated with SA, *NbPR-1a* transcript levels were approximately 10-fold higher in EV controls compared to leaves transiently expressing P6 (similar to the results shown in figure 1C). SA-treatment stimulated a further 4- to 5-fold increase in transcripts (over and above the PAMP-driven response to Agrobacterium) in both EV and P6-expressing plants. However, levels of *NbPR-1a* transcripts in SA-treated leaves that were transiently expressing P6 were still ∼6-fold lower than in SA-treated EV-plants (and only about half the level in EV-plants that had not been SA-treated). Therefore in *N. benthamiana* as with Arabidopsis, accumulation of transcripts of a representative SA-responsive marker gene was greatly reduced in leaves transiently expressing P6, although expression remained SA-responsive.

**Figure 3:**
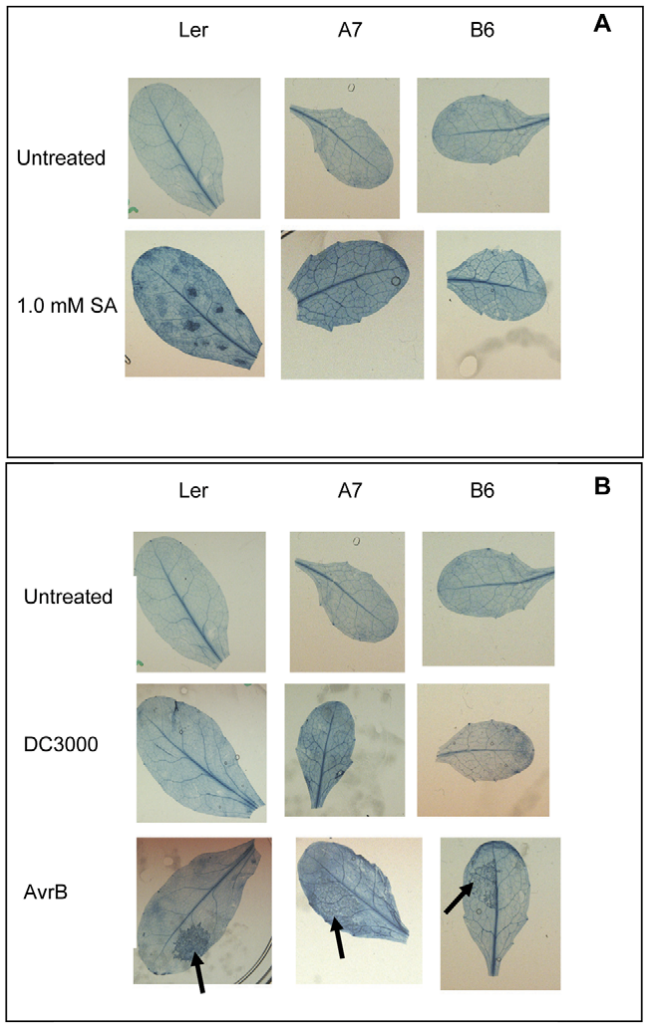
Development of necrosis (HR) on leaves of NT (L*er*) and P6-transgenic (A7 & B6) Arabidopsis. (A) Effect of SA-treatment. Panels show from top to bottom: untreated leaves and treated with 1.0 mM SA. (B) Effect of inoculation with virulent or avirulent *P*st. Panels show from top to bottom, uninfiltrated controls, leaves infiltrated with *Ps*t (DC3000) and leaves infiltrated with *Ps*t (*AvrB*). Black arrows indicate areas of leaf infiltrated with *Ps*t *AvrB* (bottom row). Necrosis was visualized by staining leaves with Trypan Blue 24 h after SA-treatment or infiltration with *Ps*t.

NT Arabidopsis also responded to SA-treatment by developing necrotic patches on leaves; these were easily identifiable 24 h after SA-treatment when the leaves were stained with Trypan Blue. In contrast P6-transgenic lines never developed necrotic lesions following SA-treatment (figure 3A). This suggests that in the presence of P6, pathways involved in the promotion of cell death in response to SA are inhibited.

### P6 expression enhances expression of JA-responsive markers

**Figure 4:**
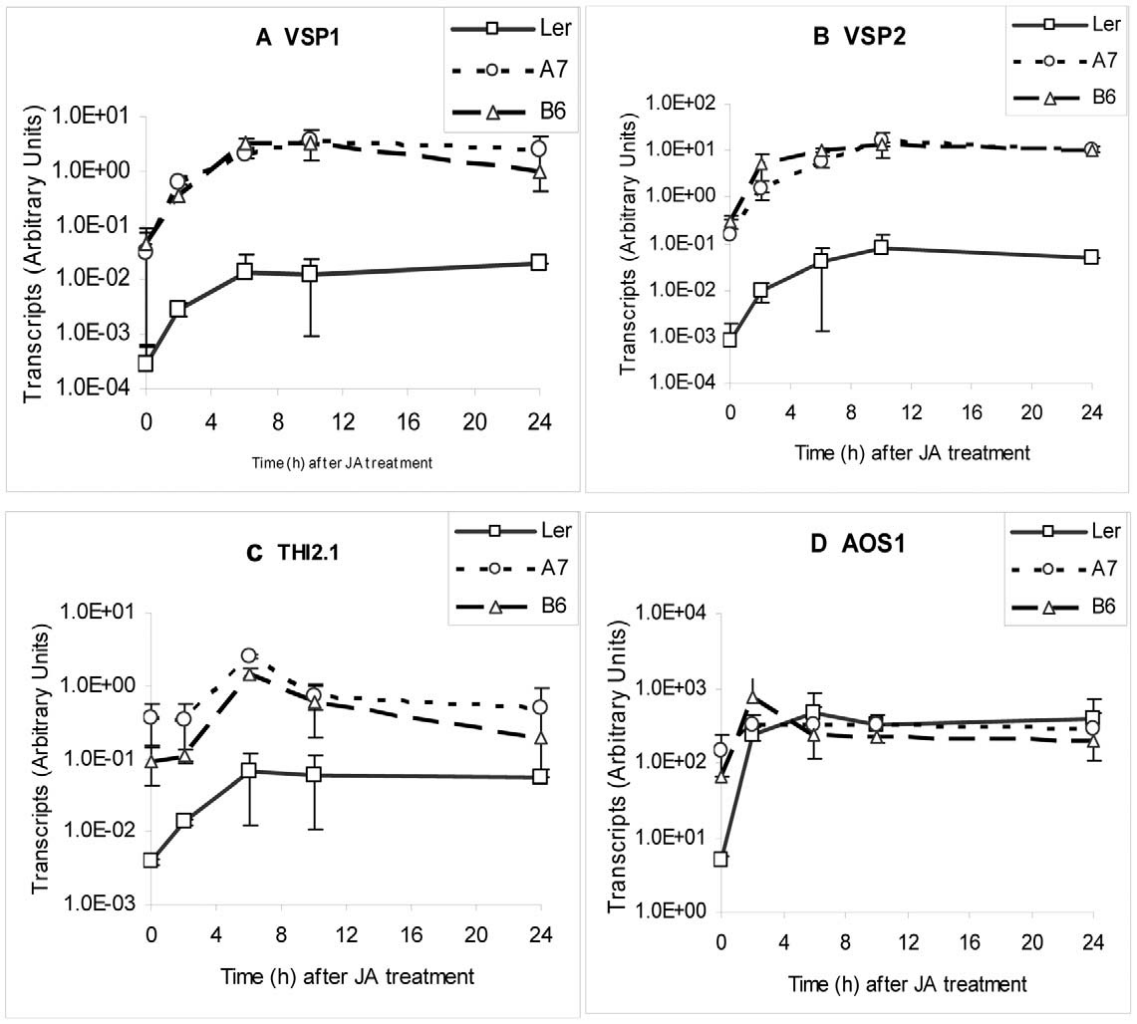
Quantification of transcripts of JA responsive genes in P6-transgenic and non transgenic plants. (A) *VSP1* (B) *VSP2*, (C) *THI2.1* and (D) *AOS1* transcript levels determined by qPCR, in P6-transgenic (A7 and B6) and non transgenic (Ler-0) Arabidopsis following treatment with 10 µM JA. Transcript levels are in Arbitrary Units and are plotted on a logarithmic scale. Each point represents mean levels of 3 independent biological samples each comprising pooled tissue from 3 plants harvested at intervals from 0 to 24 h. Error bars (positive) show standard deviations.

SA- and JA-dependent responses are coordinately but antagonistically regulated [37] and transgene-mediated expression of other VSSPs has been reported to lead to changes in the expression of JA-responsive genes [6], [38]. We therefore quantified transcripts of three representative JA-responsive marker genes *VSP1*, *VSP2* and *THI2.1*, plus *AOS1* which encodes a JA-biosynthetic enzyme. Transcripts were between 14- and 160-fold more abundant in the P6-transgenics than in NT (figure 4). To determine whether the P6-transgenic plants retained JA-responsiveness over and above the already enhanced levels of expression, we treated plants with 10 µM JA and quantified transcripts over the subsequent 24 h. JA-treatment stimulated an increase in abundance of all four transcripts both in P6 transgenic lines and NT (figure 4 – to facilitate comparison of transcript levels, data are plotted on a log scale). However, transcripts of *VSP1*, *VSP2*, and *THI2.1* were consistently ∼2 logs more abundant in the P6-transgenics than in NT throughout the 24 h after JA treatment. In contrast, with *AOS1* although JA treatment stimulated an early increase in abundance in the P6 transgenics, this was much more modest than in NT, so that by 3 h after treatment transcript levels in P6-transgenics and in NT were similar. *VSP1*, *VSP2* and *THI2.1* are all regulated downstream of JA (via the *COI1* pathway) whereas *AOS1* encodes an enzyme required for JA biosynthesis whose expression is presumably feedback-regulated by JA levels – this may account for the differences in response characteristics.

### Expression of P6 suppresses gene-for-gene and basal defences against *P. syringae*, but enhances resistance against a necrotrophic fungal pathogen

**Figure 5:**
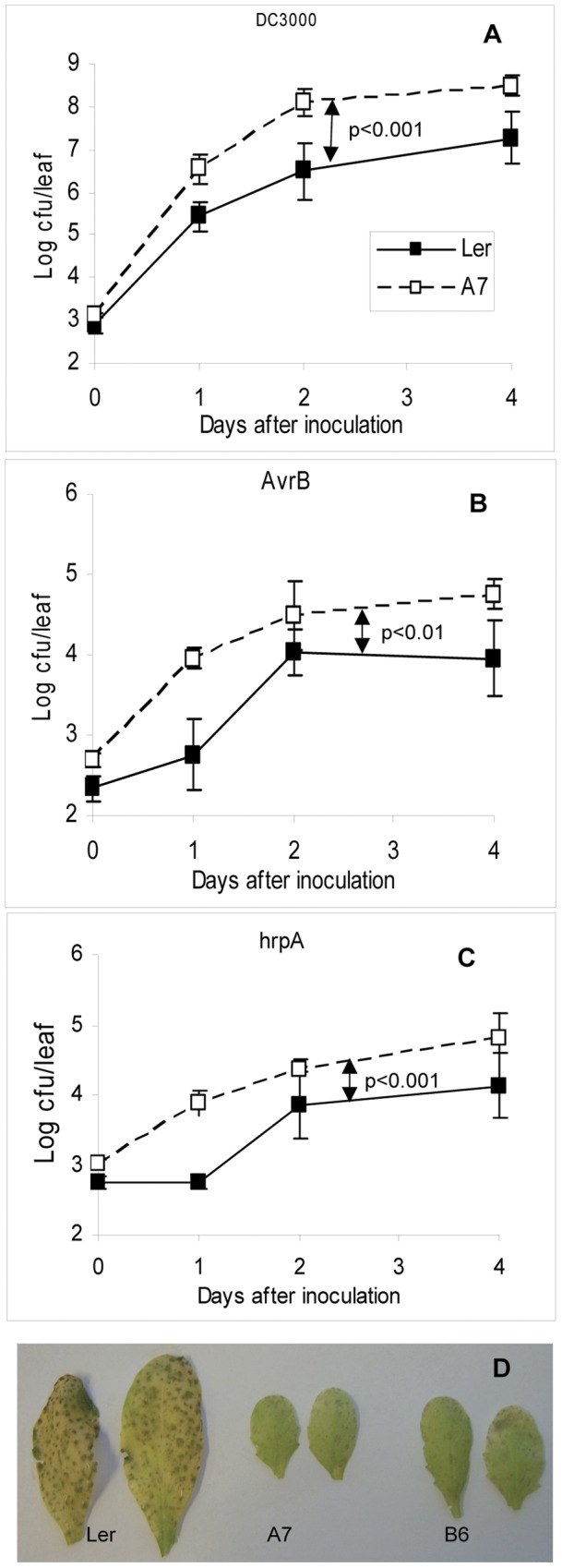
Pathogen growth in P6-transgenic (A7, B6) and NT (L*er*) Arabidopsis. (A) Titres (in cfu per leaf) of *Ps*t (DC3000) at intervals from 0 to 4 days after infiltration of leaves with 1.2×10^3^ cfu of bacteria. Titres are plotted on a log scale and show mean (± standard deviation) of colony numbers from 10 individual leaves. (B) Titres (in cfu per leaf) of *Ps*t (*AvrB*) at intervals from 0 to 4 days after infiltration of leaves with 1.2×10^3^ cfu of bacteria. Titres are plotted on a log scale and show mean and standard deviation of colony numbers from 10 individual leaves. (C) Titres (in cfu per leaf) of *Ps*t *hrpA* at intervals from 0 to 4 days after infiltration of leaves with 1.2×10^3^ cfu of bacteria. Data are plotted on a log scale and show mean and standard deviation of colony numbers from 10 individual leaves. (D) Photographs of representative leaves from non-transgenic (L*er*) and P6-transgenic (A7 and B6) plants 5 days after infection with *B. cinerea*.

SA is a central regulator of defence against biotrophic pathogens. Given the inhibitory effect of P6 on transcript levels of SA-responsive genes, we anticipated that expression of P6 might enhance susceptibility to biotrophic pathogens other than CaMV. We therefore measured growth of virulent (DC3000) and avirulent (*AvrB*) strains of *Ps*t and of the *hrpA* mutant that carries a defect in the TTSS [39] and is unable to deliver effectors into the host cell. Bacterial titres were determined in P6 transgenics and in NT at intervals up to 4 d after infiltration (figure 5). Titres of both virulent and avirulent strains were consistently 5 to 30-fold higher in P6-transgenics than in NT. The differences in titre were somewhat dependent on time after inoculation. However, multi-variate ANOVA indicated that across all three time points bacterial growth rates were very significantly higher in A7 than in NT (p<0.001 for DC3000 and p<0.01 for *AvrB*). The *hprA* mutant grew poorly in both NT and A7 but titres in the latter were still typically elevated by about 5-fold. Statistical analysis again indicated that these differences in growth were highly significant across all time points (p<0.001). As a further control, we compared bacterial growth in A7 with growth in *sid2* a mutant which lacks a functional *ISOCHORISMATE SYNTHASE 1* gene (making it unable to synthesize SA in response to infection) and exhibits a well-documented enhanced susceptibility to *Ps*t [40]. Titres of both avirulent and virulent *Pst* were significantly higher in A7 than in *sid2-2* (p<0.01), particularly for the virulent isolate DC3000 (supplementary figure S2A), suggesting that additional factors over and above the suppression of SA-signaling may be involved in the enhanced susceptibility of P6-transgenics.

P6 transgenic plants of line A7 are chlorotic and dwarfed. To eliminate any possibility that the increased susceptibility to *Ps*t might be dependent on the insertion site of the transgene or wholly or partially an indirect consequence of the dwarf phenotype rather than the inhibition of SA-signaling, we measured bacterial growth in a different P6-transgenic line B6 and also in line b2-3, a derivative of A7 that was obtained from a suppressor screen [29]. This line is homozygous for a recessive mutant allele *cse-2* at an independent locus to the transgene. The presence of *cse-2* greatly ameliorates the chlorotic dwarf phenotype of the parent A7 [29]. Compared to NT, B6 showed the same significantly enhanced susceptibility to both DC3000 and *AvrB* as did A7 (supplementary figure S2B) demonstrating that the effect is similar in two independent transgenic lines. Bacterial growth in *cse-2* (in a NT L*er* background) was similar to L*er* whereas in b2-3 (*cse-2* in an A7 background) titres were essentially identical to those in A7 (Supplementary figure S2B). Therefore, the increased susceptibility of the P6-transgenics is not attributable to the chlorotic dwarf phenotype.

Since development of SA-induced necrosis was delayed in P6-transgenic plants, we inoculated with a high titre of *Ps*t (*AvrB*) and assessed the development of HR in response to an incompatible pathogen. NT developed a fairly rapid HR, detectable by strong autofluorescence under UV-illumination 18 h after infiltration and at 24 h by a visible necrosis readily identifiable by Trypan Blue staining. In contrast, in P6 transgenic plants autofluorescence was delayed by 6–12 h and infiltrated areas stained less strongly with Trypan Blue (figure 3B) indicating that P6 expression reduces the HR triggered by a gene-for-gene interaction and delays its onset.

In contrast to biotrophs, defence against necrotrophs is regulated predominantly through JA. Since transcripts of JA-responsive markers were elevated in P6-transgenics, we inoculated plants with the necrotrophic fungus *B. cinerea* and estimated fungal growth visually (figure 5D). Compared to L*er*, both P6-transgenic lines showed visibly reduced growth of fungal hyphae and much less chlorosis at 5 dpi, indicating that they were much less susceptible to infection. This is consistent with P6 enhancing JA-signaling responses.

### P6-transgenic plants accumulate lower levels of SA, but retain pathogen-responsiveness

**Figure 6:**
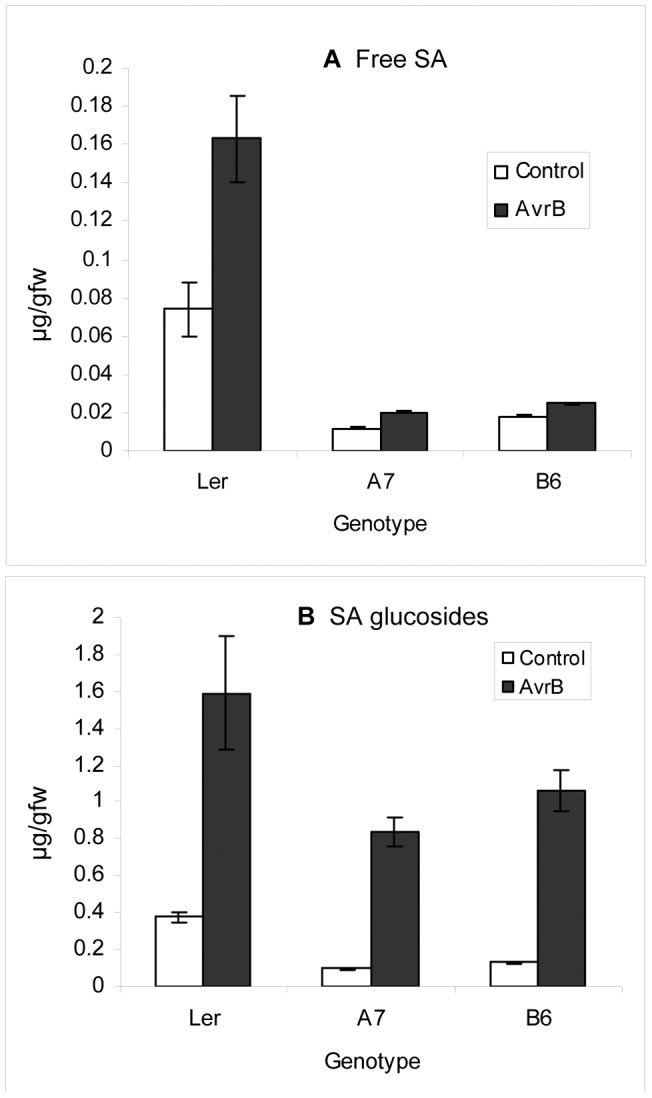
Levels of free and conjugated SA in P6-transgenic and non-transgenic Arabidopsis following inoculation with *Ps*t (*AvrB*). (A) free SA, (B) SA-conjugates (SA-β-glucoside) in leaves of NT (L*er*) and P6-transgenic (A7 and B6) plants inoculated on a single leaf with 1.2×10^3^ cfu of bacteria. Leaves from uninoculated plants were used as controls. Each sample comprised the pooled tissue from 10 leaves harvested 48 h after inoculation. Bars show mean values from 3 samples, error bars indicate standard error.

The differences in gene-expression and pathogen-susceptibility between P6-transgenic and NT plants following SA- and JA-treatments could reflect effects on SA-biosynthesis or accumulation. We measured levels of free SA and SA-β-glucoside (conjugated SA) in infected leaves of NT and P6-transgenics before and 48 h after inoculation with avirulent *Ps*t (*AvrB*) (figure 6). Concentrations of free SA in uninoculated P6-transgenics were 4-6 fold lower than in NT and levels of SA-β-glucoside were 3–4-fold lower. Infection stimulated a 1.4–1.8-fold increase in levels of free SA in P6 transgenics, and a 2.2-fold increase in NT. Levels of SA-β-glucoside in the P6-transgenics increased 10-fold following infection and although levels remained below those in infected NT, they were still much higher than in uninfected NT. Thus P6 expression reduces the overall accumulation of both free and conjugated SA in infected plants, although levels in P6-transgenic plants appeared to retain inducibility in response to a gene-for-gene interaction.

### P6 expression alters the expression and subcellular localization of NPR1

**Figure 7:**
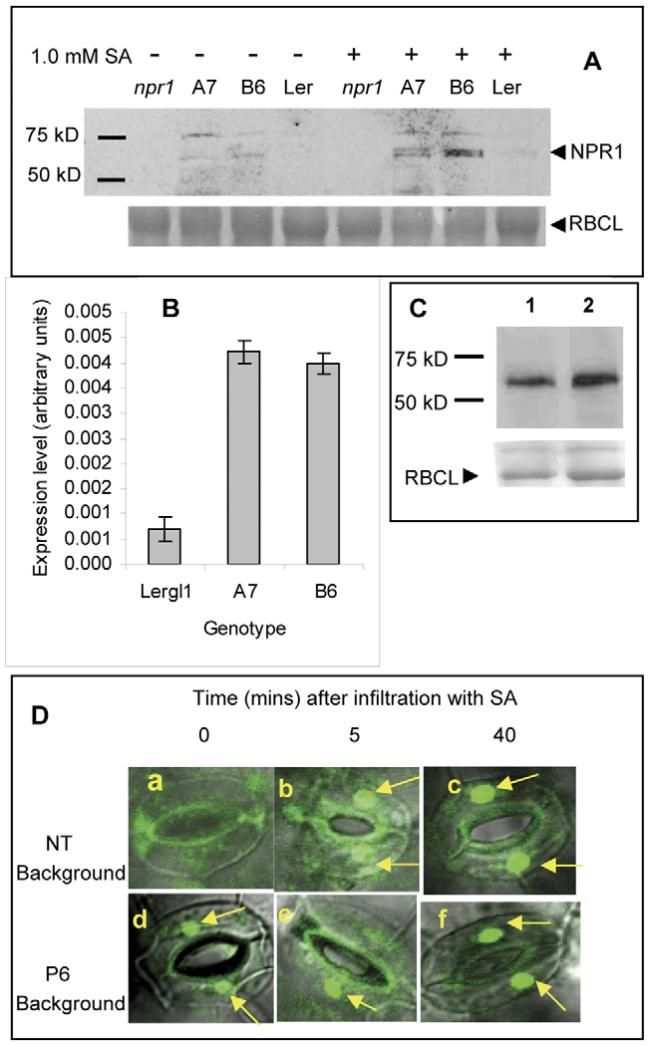
The effects of expression of P6 on NPR1. (A) Western blots of protein extracted from *npr1* mutant, P6-transgenic (A7, B6) and NT (L*er*) plants, separated by polyacrylamide gel electrophoresis. Tissue was harvested from plants either before (4 lanes on left) or 12 hours after (4 lanes on right) treatment with 1.0 mM SA. Upper panel shows blots probed with antibody to NPR1 and bands visualized by chemiluminescence. Bars on left indicate mobility of molecular weight markers; arrow indicates expected mobility of NPR1. Lower panel shows Ponceau-stained loading control; arrow indicates mobility of Rubisco Large Subunit (RBCL). (B) Western blots showing NPR1 accumulating in *N. benthamiana* leaves following agroinfiltration with a binary vector expressing NPR1 (HA-tagged at the N-terminus) under the control of a 35S promoter. Upper panel shows blots probed with anti-HA antibody and bands visualized by chemiluminescence. Lower panel shows Ponceau-stained loading control – arrow indicates mobility of Rubisco Large Subunit (RBCL). (Lane 1) HA:NPR1 co-infiltrated with empty binary vector pGWB17; (lane 2) HA:NPR1 co-infiltrated with P6 expressing binary vector pGWB-P6myc. (C) NPR1 transcript levels (in arbitrary units) determined by qPCR in P6-transgenic (A7 and B6) and NT (L*er*) plants. Bars represent mean levels of 3 independent biological samples each comprising pooled tissue from 3 plants. Error bars show standard deviations. (D) Confocal microscope images of representative pairs of guard cells from transgenic plants expressing an NPR1:GFP fusion. Panels a–c are in a NT background; panels d-f are in a P6-transgenic background (the progeny of a typical cross between the NPR1:GFP transgenic and B6). Panels a & d show samples from uninduced seedlings (infiltrated with water). Panels b & e and c & f show samples from seedlings following 5 min and 40 min (respectively) infiltration with 1.0 mM SA. Arrows indicate the nucleus.

NPR1 is a key regulator of host responses to infection by biotrophic pathogens [41], [42]. NPR1 plays a central role in regulating many of the responses to both SA and JA, notably by activating transcription of a battery of genes in response to rising SA-levels and by modulating JA responses via the COI1-dependent pathway [42], [43]. *npr1* mutants show enhanced susceptibility to both virulent and avirulent strains of *Ps*t and accumulate very low levels of *PR-1* and *BGL2* transcripts in response to infection or SA-treatment [44]. The similarities between these aspects of the phenotypes of *npr1* mutants and P6 transgenic lines suggested a potential involvement for NPR1 in the action of P6. We therefore compared levels of NPR1 in P6-transgenic and NT plants in western blots probed with an anti-NPR1 antibody (figure 7A). A band of ∼60 kD corresponding to the expected size of NPR1 was visible in P6-transgenics and very faintly in NT plants. Following treatment with SA the relative intensity of the band was increased in both transgenic and NT plants, but it remained much stronger and appeared as a doublet in the P6-transgenic lines. We assume that the band(s) must correspond to authentic NPR1 since they were undetectable in extracts from a null mutant *npr1-1* even after SA-treatment.

To determine whether increased levels of NPR1 protein might be a result of greater transcript levels we measured *NPR1* transcripts by qPCR and found that they were approximately 7-fold more abundant in P6-transgenics than in L*er* (figure 7B). To determine whether P6 might be acting at the protein level, e.g. by stabilizing NPR1 against proteolysis, we transiently co-expressed HA-tagged NPR1 and either P6myc or as a control EV (pGWB17) in *N. benthamiana*. NPR1 levels were quantified in western blots using an anti-HA antibody (figure 7C). The abundance of NPR1:HA was unaltered when co-expressed with P6 compared to EV co-expression controls; therefore P6 does not appear to be affecting protein stability. Most likely the greater levels of NPR1 when P6 is present are a consequence of enhanced gene-expression.

NPR1 undergoes a redox-dependent translocation from cytoplasm to nucleus in response to increasing concentrations of SA; it is the presence of activated NPR1 in the nucleus that activates transcription of genes of the *PR1* regulon [45]. To investigate whether P6 might be affecting the localization and/or activation of NPR1, we used a transgenic reporter line that constitutively expresses a NPR1:GFP fusion [46] to monitor its subcellular location before and after SA-treatment. Under our growth conditions, mature *35S::NPR1:GFP* transgenic plants were dark green and dwarfed with necrotic micro-lesions, a phenotype reminiscent of mutants with constitutively activated SA-mediated responses e.g. *cpr5* [47]. Progeny of crosses between *35S::NPR1:GFP* and either of the P6-transgenic lines were larger than both parents and did not develop necrotic lesions. Evidently the P6 transgene suppresses the *cpr5*-like phenotype which presumably results from constitutive overexpression of NPR1:GFP. In turn, the latter suppresses the chlorotic dwarf phenotype which results from ectopic overexpression of P6.

Cotyledons from soil-grown seedlings were infiltrated with 1 mM SA to induce defence responses and examined by confocal microscopy to determine the subcellular location of NPR1:GFP. Uninduced controls were infiltrated with water. figure 7D shows representative images of guard cells (as used to show nuclear and cytoplasmic localization of NPR1:GFP by Mou *et* *al*. [46]). In uninduced plants with the wild-type background, fluorescence was visible in the cytoplasm with no obvious accumulation in the nuclei. Following 5 min treatment with SA, much of the fluorescence was now located in the nuclei, and by 40 min nuclei appeared strongly fluorescent (figure 7D panels a, b and c). To confirm that the site of NPR1 localization was indeed in nuclei, we carried out DAPI (4′,6-diamidino-2-phenylindole) staining and observed colocalization of GFP and DAPI fluorescence in SA-induced plants (supplementary figure S3). When we repeated the infiltrations on several independent progeny of crosses between *35S::NPR1:GFP* and either A7 or B6 (i.e. seedlings containing both P6 and *35S::NPR1:GFP* transgenes), the nuclei always exhibited strong GFP fluorescence even in the uninduced (water-infiltrated) leaves (panel d); SA-treatment had no obvious further effect on its location (panels e and f). We conclude that P6 stimulates the accumulation of enhanced amounts of NPR1, which are apparently targeted to the nucleus even in uninduced plants (although presumably in a form that cannot stimulate transcription of *PR* genes).

## Discussion

Expression *in planta* of P6, a pathogenicity determinant encoded by CaMV, strongly inhibited SA-dependent responses including the expression of representative pathogenesis-related genes, hypersensitive cell death, and basal defence against biotrophic bacterial pathogens. We have previously showed that long distance movement of CaMV is almost completely inhibited in Arabidopsis mutants that accumulate high levels of SA and show constitutively activated SAR [10]. Therefore SA-dependent defence responses, in particularly when they are pre-engaged (as in these mutants), must be capable of restricting long distance movement of CaMV. During natural CaMV-infections of wild-type plants an effect of the accumulation of P6 in infected leaves might be to suppress the activation of these SA-dependent defence responses thereby facilitating systemic spread.

P6 is a multifunctional protein one of whose activities is suppressing RNA silencing [26], [27]. In addition to affecting SA-responses, P6 also had a profound effect on JA-signaling, strongly up-regulating representative JA-responsive marker genes. SA- and JA-signaling pathways are regulated in a mutually antagonistic manner through mechanisms that at least partially rely on the activity of NPR1 [37], [43]. There have been several reports of VSSPs modifying JA-responsive gene-expression. Transgene-mediated expression of two geminivirus VSSPs C2 and βC1 suppressed JA-dependent responses, albeit by different mechanisms [48], [49]. HC-Pro from a potyvirus and 2b from CMV had profound effects on the patterns of expression of JA-responsive genes in their hosts [6], [38], [50]. However, in contrast to P6, none of these VSSPs significantly altered the global patterns of expression of SA-responsive genes and they seemed to be more specifically targeted at JA-signaling. P6 therefore appears to be unique in this respect, being the prototype member of a novel class of pathogenicity effectors that that directly targets SA-signaling. P6 accumulates to high levels during CaMV infection [13], [28] providing a plausible explanation for our previous observation that in individual leaves, virus invasion coincides with a *decrease* in the abundance of transcripts of SA-responsive genes [15].

Transgene-mediated expression of VSSPs can have pleiotropic effects on developmental and stress responses. Endres *et* *al*. [38] suggested that the global activation of wounding-, JA-, cold- and heat-responsive genes by potyvirus HC-Pro might indicate a compensatory relationship between RNA-silencing and stress- and defence-response pathways. However, three other VSSPs CMV 2b and the geminivirus-encoded C2 and βC1, all *suppressed* JA-responsive gene expression [6], [48], [49], exactly the opposite effect to HC-Pro (and P6). Therefore, it is probably an oversimplification to directly link suppression of silencing with a general up-regulation of stress and defence responses.

P6 plays an essential role in CaMV replication by facilitating the non-canonical translation of downstream ORFs on the 35S RNA [13]. Since P6^Y305P^, a mutant form that is defective in TAV activity, suppressed PAMP-responsive expression of *NbPR-1a* as efficiently as wild-type P6, TAV activity (which localizes to the central region of P6) does not appeared to be required for the suppression of SA-signaling. Replication (TAV) and pathogenicity functions may therefore be at least partially separated. Recent work in our laboratory (C. Carr, J. Laird and JJ Milner, unpublished data, 2012) indicates that sequences that are essential for both of the pathogenicity functions (suppression of silencing and suppression of *PR1a* expression) map within the N-terminal 112 amino acid domain. Thus the two activities may be functionally linked. Kobayashi and Hohn [51], [52] introduced in-frame deletions to the same N-terminal domain resulting in virus mutants that were replication-competent but deficient in long-distance movement – again this is consistent with our proposed link between SA-dependent defence responses and long-distance movement of CaMV. The N-terminal region of P6 is an avirulence domain in some Arabidopsis ecotypes and in at least two *Nicotiana* species [20], [24], [25], consistent with the zig-zag model for the evolution of defence mechanisms in plants [53] whereby effectors that suppress basal defence themselves become targets for effector-triggered immunity.

P6 interacts directly with nuclear protein DRB4 and is believed to suppress RNA-silencing by modifying DICER activity [27]. If silencing suppression and SA-signaling suppression are linked, it is not clear which activity underlies the other. Shivaprasad *et* *al*. reported that P6 interferes with RDR6-dependent siRNA and tasiRNA pathways [54], and that small RNA populations undergo global changes during CaMV-infection [54]–[56]. Some of these small RNAs might be involved in regulating SA- and JA-signaling thereby providing a mechanism through which P6 might suppress SA-signaling. This hypothesis is consistent with our observation that in the presence of P6, despite overall levels of SA- and JA-responsive transcripts being profoundly affected, responsiveness to exogenous application of SA or JA was still present. An alternative model puts SA-signaling as the primary target for P6, silencing suppression perhaps being a secondary consequence of an effect on the SA-responsive RNA-amplification loop. RDR1, an SA-responsive RNA-dependent RNA polymerase [57] is a potential link between silencing and SA-mediated defence [11]. However although RDR1 plays an important role in the generation of siRNAs in CMV-infected plants [58], [59], RDR6, whose expression is not SA-responsive, may be the more important target for P6 [54].

Sequence analysis of P6 predicts several putative functional domains (e.g. RNA-binding, Zn-finger) but no homologues are known outside the closely related members of the Caulimovirus and Soymovirus families of the *Caulimoviridae*. Apart from these small groups of viruses, the TAV-dependent translation of a polycistronic mRNA has no obvious equivalent elsewhere in eukaryotes. Although TAV and defence suppression domains may have evolved independently and their evolutionary lineage remains obscure, P6 appears to be a functionally and structurally unique protein.

Translocation of NPR1 from cytoplasm to nucleus is a critical step in the transcriptional activation of genes of the *PR-1* regulon, so our finding that NPR1 is both more abundant and more strongly nuclear-localized in the presence of P6 was unexpected and at first sight counter-intuitive. However *35S::NPR1:GFP 35S::P6* double transgenics do not show the “constitutively-activated-defence” phenotype of the *35S::NPR1:GFP* parent: *PR-1* levels are low and the dwarf lesion-mimic phenotype is absent. Therefore, notwithstanding its localization to the nucleus, when P6 is present NPR1 cannot be functioning normally as a transcriptional activator.

At low intracellular SA levels NPR1 exists predominantly in the cytoplasm as an inactive multimer [45], [60]. Increasing concentrations of SA promote monomerization and activation of NPR1 which is translocated into the nucleus where it interacts with bZIP transcription factors stimulating transcription of genes in the *PR-1* regulon [45]. Additional layers of complexity are added to the model by roles for phosphorylation and S-nitrosylation of NPR1 and for its turnover by targeted proteolysis [60], [61]. In addition to its nuclear functions, NPR1 modulates cross-talk between JA- and SA-signaling, acting in the cytoplasm to regulate JA-signaling via the *COI1* pathway [45]. Active NPR1 is also essential for SA-accumulation [62] providing a plausible explanation for the low endogenous levels of SA in P6-transgenics.

Even in normal uninduced cells, small amounts of an inactive form of NPR1 are translocated into the nucleus; this is recruited to the *PR-1* promoter but cannot activate transcription [60]. Perhaps this nuclear localization of inactive NPR1 might be stimulated in the presence of P6, greatly reducing the expression of genes in the *PR-1* regulon. Unlike *PR-1* and *BGL2*, *AOX1a* is not thought to be regulated via NPR1. The much smaller effect of P6 on levels of transcripts of *AOX1a* compared to *PR-1* and *BGL2* is consistent with this hypothesis, and suggests that alternative signaling pathways may also be involved. To date we have been unsuccessful in attempts to demonstrate a direct interaction between P6 and NPR1 either in yeast or by immune co-precipitation or by an association *in planta* (A. Love, C. Geri, C. Carr and J. J. Milner, unpublished data, 2010/11). Therefore, an indirect mechanism, perhaps involving a role for siRNAs or miRNAs, appears more probable. Whatever the mechanisms of action, P6 should prove a valuable tool with which to uncover novel components (or novel roles for existing ones) that might regulate the activity of NPR1 during SAR.

SA is the key regulator of plant defence against biotrophic pathogens [63]. It is therefore unsurprising that P6-transgenic Arabidopsis show markedly enhanced susceptibility to both avirulent and virulent *Ps*t (and enhanced resistance to a necrotrophic pathogen). Indeed, increased susceptibility extends to *Pseudomonas* species to which Arabidopsis is normally a non-host (C. Geri, A. Love, J. Laird, M. Tunney and J. Milner, unpublished data, 2009). If these phenomena extend to crop species, CaMV-infected crop plants are likely to be more susceptible to opportunistic infection by a whole range of biotrophic pathogens and this knock-on effect would make CaMV a much more economically important pathogen than previously realized.

## Materials and Methods

Transgenic P6-expressing Arabidopsis lines A7 and B6 have been described [28], [29]. Transgenic Arabidopsis expressing NPR1:GFP from a 35S promoter [46] were a gift from Prof Xinian Dong (Duke University, USA). Non-transgenic (NT) plants were L*er*-0 (unless stated otherwise). Inoculation with CaMV (isolate Cabb B-JI) was carried out according to Cecchini *et* *al* [64]. Plants were inoculated with *P. syringae* by infiltrating 1.2×10^3^ cfu of bacteria according to Grant *et* *al*. [14], [65]. Results were analyzed statistically by Multi-Variate Analysis of Variance (MANOVA) as described by Love *et* *al*. [14]. Necrotic lesions were assessed by Trypan Blue staining following infiltration of 1.2×10^5^ cfu [66]. Infection with *B. cinerea* was carried out according to Grant *et* *al*. [67].

Transient expression was obtained by infiltrating *N. benthamiana* leaves with *A. tumefaciens* GV3101 carrying the appropriate expression vector according to Bazzini *et* *al* [68]. The P6-expression vector pJO-BJI and the parent Ti binary vector pJO530 are as described [28], [29]. Details of pGWB-P6^Y305P^, pGWB-P6myc and pGWB-HA:NPR1 are given in SI.

SA and JA treatments were carried out by spraying plants with 1.0 mM SA or 10 µM JA using a hand-held mister as described by Love *et* *al*. [14]. RNA was extracted from tissue samples, checked for integrity and transcripts were measured by qPCR using a Stratagene MX4000 thermocycler. PCR conditions, protocols and analysis of the data have been described previously [10], [14].

Free SA and SA-β-glucoside concentrations were determined using a mini-scale procedure based on high pressure liquid chromatography [67].

Fluorescence microscopy was carried out using a Zeiss LSM510 confocal microscope as described previously [10].

Full details of methods are given in Methods S1.

## Supporting Information

Figure S1 **Accumulation of P6 protein and transcripts in** ***N. benthamiana*** **leaves following agroinfiltration.** (E) (A) Western blots showing levels of P6 protein. Tissue was collected 3 days after infiltration. (Lane 1) uninfiltrated *N. benthamiana* leaves; (lane 2) leaves infiltrated with Agrobacterium carrying empty vector pJO530; (lane 3) leaves infiltrated with Agrobacterium carrying P6 expression vector pJO-BJI; (lane 4) P6 transgenic Arabidopsis line A7 (tissue collected from 3 week old plants). Top panel shows blots probed with anti-P6 antibody and bands visualized by chemiluminescence. Bars on left indicate mobility of molecular mass markers, arrow indicates expected mobility of P6. Bottom panel shows Ponceau stained loading control; arrow indicates Rubisco Large Subunit (RBCL) (F) (B) P6 transcripts, determined by qPCR, in *N. benthamiana* leaves harvested 48 h after agroinfiltration. Leaves were infiltrated with Agrobacterium carrying the following binary plasmids (EV) pJO530, (pJO-BJI) pJO-BJI, (P6myc) pGWB-P6myc, (P6Y305P) pGWB-P6Y305P. Bars show mean levels (in arbitrary units) of 3 independent biological samples each comprising 3 pooled infiltrated leaf sections. Error bars show standard deviations.
(TIF)

Figure S2 **Titres of** ***Ps*** **t DC3000 and** ***AvrB*** **in wild-type, mutant and P6 transgenic Arabidopsis lines.** Colony counts were carried out on leaves harvested 54 h after inoculation with 1.2×10^3^ cfu. Bars show mean of 10 individual leaves, error bars show standard deviations. (A) Col-0, *sid2-2* and A7. (B) L*er*, A7, B6, *cse-2* mutant in L*er* background and *cse-2* mutant in a P6 (A7) background (line b2–3).
(TIF)

Figure S3 **Nuclear localization of NPR1:GFP.** Confocal microscope images of a representative pair of guard cells from transgenic plants expressing an NPR1:GFP fusion infiltrated with 1.0 mM SA and stained with DAPI. Panels show from left to right GFP fluorescence (rendered in green) DAPI fluorescence (rendered in blue) and the merged images.
(TIF)

Table S1 **Primer sets used in Real-Time qPCR.**
(DOC)

Methods S1 (DOC)
