# Supplementary figures and images for: Cauliflower mosaic virus Protein P6 Inhibits Signaling Responses to Salicylic Acid and Regulates Innate Immunity
Source: PLoS One. 2012 Oct 11;7(10):e47535. doi: 10.1371/journal.pone.0047535 (PMC3469532; doi:10.1371/journal.pone.0047535)

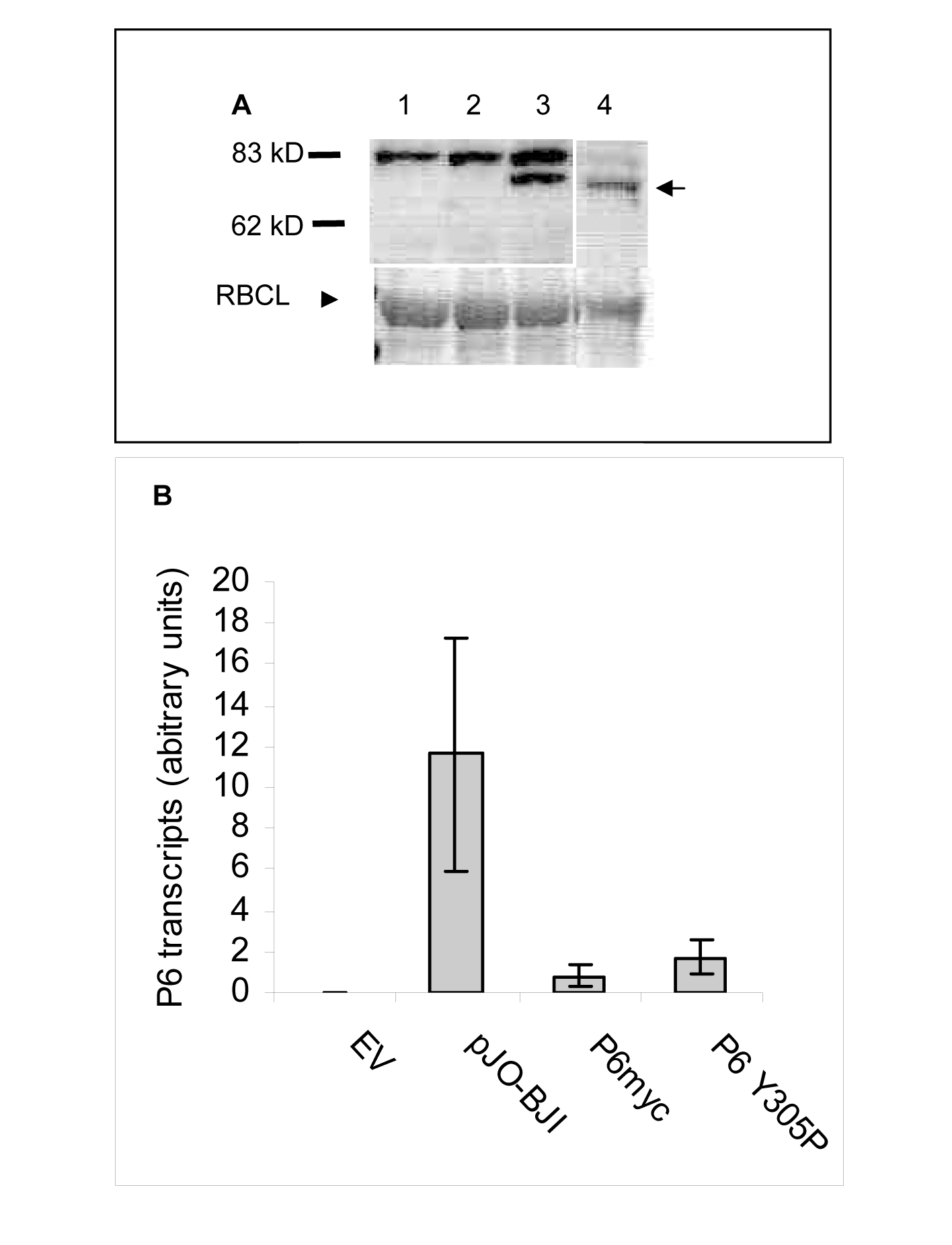

Supplement: Figure S1 — Accumulation of P6 protein and transcripts in N. benthamiana leaves following agroinfiltration. (E) (A) Western blots showing levels of P6 protein. Tissue was collected 3 days after infiltration. (Lane 1) uninfiltrated N. benthamiana leaves; (lane 2) leaves infiltrated with Agrobacterium carrying empty vector pJO530; (lane 3) leaves infiltrated with Agrobacterium carrying P6 expression vector pJO-BJI; (lane 4) P6 transgenic Arabidopsis line A7 (tissue collected from 3 week old plants). Top panel shows blots probed with anti-P6 antibody and bands visualized by chemiluminescence. Bars on left indicate mobility of molecular mass markers, arrow indicates expected mobility of P6. Bottom panel shows Ponceau stained loading control; arrow indicates Rubisco Large Subunit (RBCL) (F) (B) P6 transcripts, determined by qPCR, in N. benthamiana leaves harvested 48 h after agroinfiltration. Leaves were infiltrated with Agrobacterium carrying the following binary plasmids (EV) pJO530, (pJO-BJI) pJO-BJI, (P6myc) pGWB-P6myc, (P6Y305P) pGWB-P6Y305P. Bars show mean levels (in arbitrary units) of 3 independent biological samples each comprising 3 pooled infiltrated leaf sections. Error bars show standard deviations. (TIF) [file pone.0047535.s001.tif]

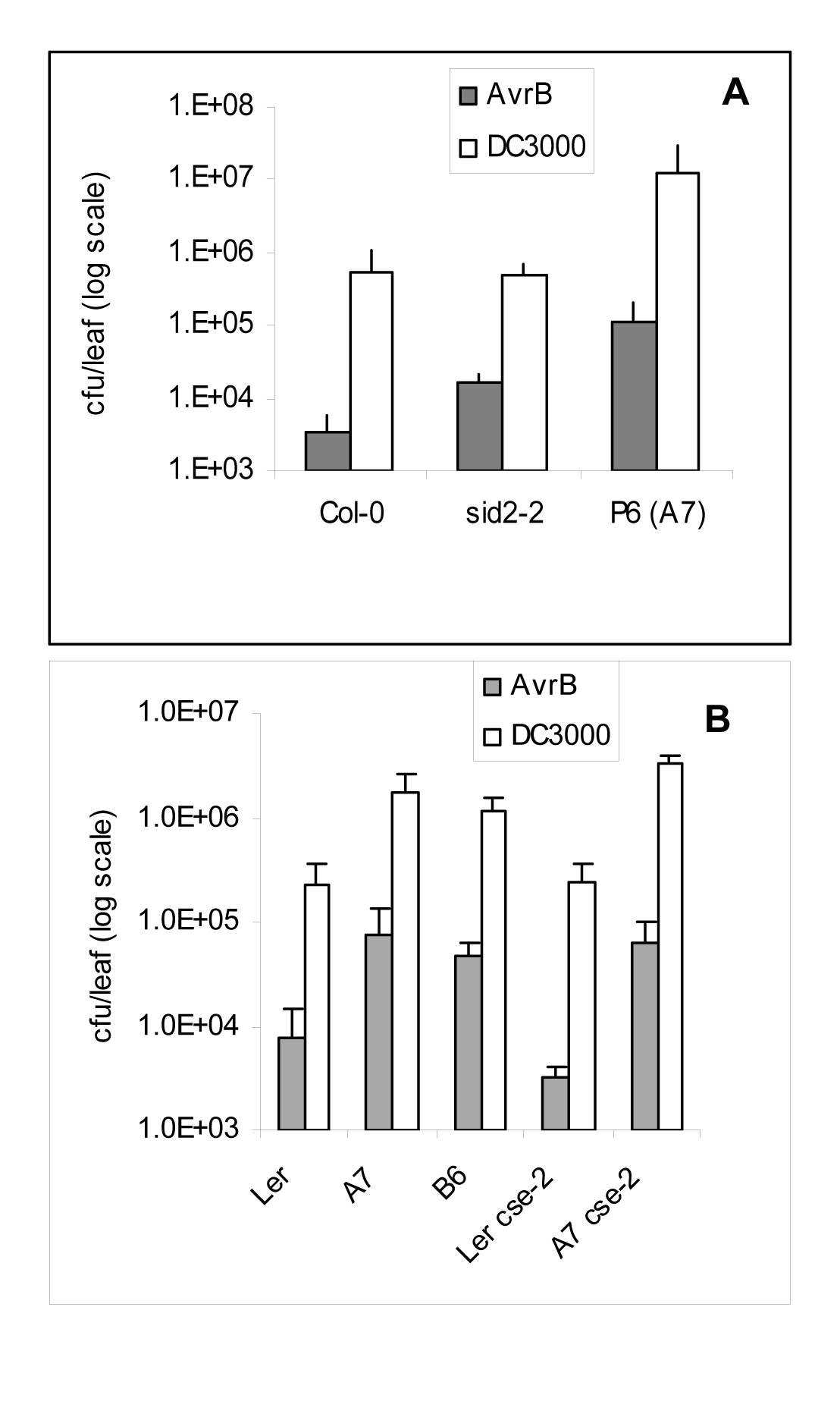

Supplement: Figure S2 — Titres of Ps t DC3000 and AvrB in wild-type, mutant and P6 transgenic Arabidopsis lines. Colony counts were carried out on leaves harvested 54 h after inoculation with 1.2×10^3^ cfu. Bars show mean of 10 individual leaves, error bars show standard deviations. (A) Col-0, sid2-2 and A7. (B) Ler, A7, B6, cse-2 mutant in Ler background and cse-2 mutant in a P6 (A7) background (line b2–3). (TIF) [file pone.0047535.s002.tif]

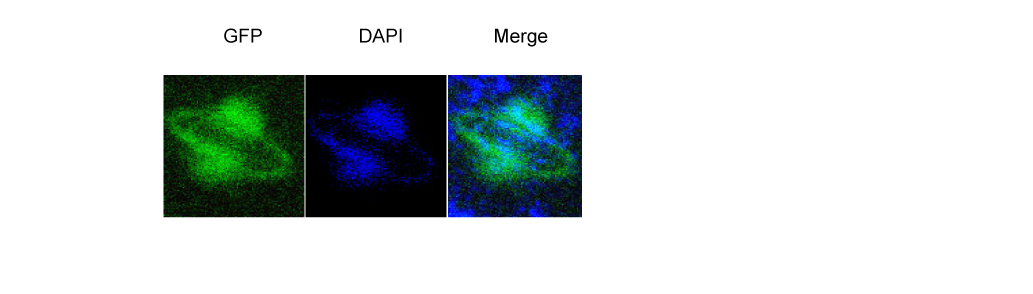

Supplement: Figure S3 — Nuclear localization of NPR1:GFP. Confocal microscope images of a representative pair of guard cells from transgenic plants expressing an NPR1:GFP fusion infiltrated with 1.0 mM SA and stained with DAPI. Panels show from left to right GFP fluorescence (rendered in green) DAPI fluorescence (rendered in blue) and the merged images. (TIF) [file pone.0047535.s003.tif]
