# Supplementary material for: Cauliflower mosaic virus Protein P6 Inhibits Signaling Responses to Salicylic Acid and Regulates Innate Immunity
Source: PLoS One. 2012 Oct 11;7(10):e47535. doi: 10.1371/journal.pone.0047535 (PMC3469532; doi:10.1371/journal.pone.0047535)
Supplement: Table S1 — Primer sets used in Real-Time qPCR. (DOC) [file pone.0047535.s004.doc]

**Table S1: Primer sets used in Real-Time qPCR.**

| Gene | Forward Primer | Reverse Primer |
| --- | --- | --- |
| *AtACT2* | CTAAGCTCTCAAGATCAAAGGCTTA | ACTAAAACGCAAAACGAAAGCGGTT |
| *AtAOX1A* | CTGGAGCTTCCTTTAGTTCA | ACATTGAGAATGTTCCTGCT |
| *AtPR-1* | TCAGTGAGACTCGGATGTG | CCTGCATATGATGCTCCTT |
| *AtBGL2* | CCTATTCGACGCAAATCTC | CTTCTCGGTGATCCATTCT |
| *AtAOS1* | CGATTTCTCTCCACCCAAAA | ATCCCAACGGTCTTTGATTG |
| *ATVSP1* | AGTTTCAAGAGGTTTTCG TA | CAATACGACTCC AAAACTGTA A |
| *AtVSP2* | CCATTAGGCTTCAATATGAGAT | AAATATGGATACGGGACAGAG |
| *AtTHI2.1* | AATGGAGCGTCGGAACAA | CGACACATGCACACACACA |
| *AtNPR1* | GAAGCACACCTGCAGCAATA | TCGGTGAGACTCTTGCCTCT |
| *NbEF1α* | TTGGAAATGGATATGCACCA | GCAAAGGTCTCCACAACCAT |
| *NbPR1a* | ACTCTTGTCGTGCCCAAAAT | GTTTTCGCC GTATTGACCAT |
| 35S RNA | TAC GCC AAC TTC GAC TCT CA | TGC TCG CTT TGG GTA TTT TC |
| CaMV P6 | GCT CCT GGT AAA GAA TCA AC | GTG TTG AAT ACC CGA TTC TG |
