# Supplementary material for: Cauliflower mosaic virus Protein P6 Inhibits Signaling Responses to Salicylic Acid and Regulates Innate Immunity
Source: PLoS One. 2012 Oct 11;7(10):e47535. doi: 10.1371/journal.pone.0047535 (PMC3469532; doi:10.1371/journal.pone.0047535)
Supplement: Methods S1 — (DOC) [file pone.0047535.s005.doc]

**SUPPORTING INFORMATION**

**Materials and Methods**

*Plants and Growth Conditions:*

Arabidopsis plants were grown in compost in a controlled environment at a temperature of 22°C. Light was provided by Osram “Warm White” fluorescent tubes at an intensity of 100 μmols.m^-2^.s^-1^ for 10 hours per day. P6-expressing transgenic lines A7 and B6, which are in a L*er* *gl1* background, have been described elsewhere [1]. They contain a single copy of the Gene VI coding region from CaMV Cabb B-JI under the control of a CaMV 35S promoter. Transgenic line b2-3, which was identified in a suppressor mutant screen, was derived from A7 and contains the *35S:P6* transgene together with a recessive mutation at a single locus, *cse*, which substantially suppresses the chlorotic dwarf phenotype of A7. The *cse-2* mutant line in a non transgenic (L*er*) background was obtained by crossing out the gene VI transgene. These lines have been described in detail elsewhere [2]. Transgenic Arabidopsis lines expressing an NPR1:GFP fusion have been described by Mou *et al*. [3]. Expression of the transgene is under the control of a 35S promoter.

*Nicotiana benthamiana* plants were grown in a similar environment, but under 16 h day length and at a light intensity of 200 μmols.m^-2^.s^-1^.

*Transient Gene Expression in* N. benthamiana*.*

Plants were infiltrated with *A. tumefaciens* GV3101 carrying the appropriate Ti binary expression vector according to Bazzini *et al.* [4]. Overnight bacterial cultures were diluted to an OD_600_ of 0.2 with 10 mM MgCl_2_. Acetosyringone was added to a concentration of 200 μM and after 2 hours bacteria were pressure inoculated into the undersides of leaves. Expression of P6 was ordinarily obtained using the binary Ti plasmid pJO-BJI [1] in which the coding region of P6 from CaMV Cabb B-JI is under the control of a 35S promoter. In some experiments expression of P6 was obtained using binary Ti plasmids pGWB-P6myc which contains the same P6 coding sequence as pJO-BJI (minus the stop codon) inserted into the Gateway binary vector pGWB17 [5] giving 35S promoter-driven expression of P6 fused at the C-terminus to a 4 x myc tag. Expression of P6^Y305P^ was obtained using the same Gateway binary vector but expressing a P6 variant in which the Tyrosine at amino acid 305 has been substituted with Proline. Transient expression of NPR1 was obtained using the binary Ti vector pGWB-HA:NPR1. This comprises the complete coding sequence of NPR1 from Arabidopsis Col-0 inserted into the Gateway binary vector pGWB15 [5] to obtain expression of NPR1 fused to a 3 x HA tag with expression driven by a 35S promoter.

*Infection with Pathogens.*

For infection with virus, following the emergence of the first true leaves Arabidopsis seedlings were manually inoculated on single leaf with 2 μL of water containing 100 ng of purified CaMV (isolate Cabb B-JI), as described by Cecchini *et al.* [6]. Bacterial infections were carried out according to Grant *et al*. [7]. Overnight cultures of *Ps*t DC3000, *AvrB* or *hrpA* were resuspended in 10 mM MgCl_2_ at an OD_600_ of 0.2 (equivalent to 10^8^ cfu.ml^-1^) and further diluted to 2 x 10^5^ cfu/ml. Plants were manually inoculated on a single leaf using a syringe by pressure-infiltrating 6 μl of bacterial suspension (equivalent to 1.2 x 10^3^ cfu). The *hrpA* mutant, which lacks a functional TTSS [8], was a kind gift from Prof Murray Grant (University of Exeter, UK). Infected leaves were detached at appropriate intervals, ground in 10 mM MgCl_2_ and appropriate dilutions were plated out for colony counting according to Grant *et al.* [7]. Data were analyzed statistically by multivariate ANOVA using the Bonferroni method [9]. For assessment of the development of necrotic lesions plants were inoculated by infiltrating 6 μl of bacterial suspension as above but at a concentration giving an inoculum of 1.2 x 10^5^ cfu. After 24 h leaves were stained with Trypan Blue according to Glazebrook and Weigl [10]. Infection with *B. cinerea* was carried out by spraying plants with a suspension of fungal spores in water according to Grant *et al.* [11]. Disease symptoms were assessed and plants photographed after 5 d.

*Quantification of Transcripts by qPCR:*

Transcript levels in Arabidopsis were quantified by Real Time RT-PCR using the Stratagene MX4000 Real-Time thermocycler and the data were analyzed as described by Love *et al.* [9,12]. RNA was extracted from approximately 50 mg of tissue [9], RNA samples (50 ng) were checked for integrity by agarose gel electrophoresis and transcribed to cDNA in 20 μl reactions using Qiagen Sensiscript kits (Qiagen, Crawley, UK) according to the manufacturer’s protocol. cDNA was quantified using Brilliant Kits (Agilent, Wokingham, UK). Reactions were carried out in duplicate for each biological sample which comprised cDNA derived from the pooled RNA from 3 individual plants. *ACT2*  was used as an internal reference standard [9]. Primers used are detailed in Table S1. The protocols (primer design, optimization of qPCR conditions) have been optimized and carried out in accordance with MIQE guidelines [13].

Transcript levels in *N. benthamiana* were determined by Real Time RT-PCR essentially as above except that each biological sample comprised RNA extracted from approximately 50 mg of tissue taken from the area of a single leaf infiltrated with Agrobacterium. A 700 bp fragment of *NbPR1a* was amplified using a set of universal primers from Dean *et al.* [14] and the sequence used to design primers for qPCR. *EF1α* was used as an internal reference standard [14]. qPCR primers are detailed in Table S1.

*Analysis of Proteins in Western Blots.*

Proteins were separated by electrophoresis in 10% polyacrylamide gels and transferred to nylon membranes by electroblotting using standard procedures. Bands were visualized using Millipore Immobilon ECL kits from Fisher Scientific (Loughborough, UK). Antibodies against CaMV P6 were developed against isolate W260 [15] and were a kind gift from Prof J. Schoelz (University of Missouri, USA). Anti-HA and anti-myc antibodies were purchased from NEB (Hitchin, UK). Polyclonal antibodies against NPR1 were raised against a peptide comprising the N-terminal 246 amino acids. Antibodies were purified from serum by affinity chromatography as described by Mou *et al.* [3]. Details of the anti-NPR1 antibodies are given in Spoel *et al* [16].

*Treatment with Salicylic Acid and Jasmonic Acid:*

A hand held mister was used to spray plants with 1.0 mM SA or 10 μM JA in 10 mM Na phosphate buffer, pH 7.0. For intracellular localization of NPR1:GFP, cotyledons were detached and infiltrated with 1.0 mM SA or water under negative pressure in a syringe barrel before mounting on a microscope slide for confocal microscopy.

*Assay of Free and Conjugated SA.*

Briefly, 200mg of inoculated leaf tissue from 5 weeks old plants was collected 48 h after infiltration with *Ps*t *AvrB,* ground in liquid nitrogen, extracted in 90-100% ethanol and vacuum dried. The resulting pellet was resuspended in 5% (w/v) trichloroacetic acid and mixed with ethylacetate:cyclopentane:isopropanol (50:50:1) solution. Fractionation of this resuspension by simple centrifugation provided free SA (upper part) and SA-β-glucoside (lower part) fractions. The final filtered samples (50μl per injection) were subjected to HPLC (DIONEX, UK) equipped with a C-18 column (250x4.6mm, Phenomenex, UK). Methanol and acetic acid were used for elution.

*Fluorescence microscopy:*

Localization of NPR1:GFP was followed using a Zeiss LSM510 confocal microscope essentially as described in Love *et al* [12]. For GFP fluorescence the excitation wavelength was 488 nm and the emission filters were set for the range 505 to 530 nm. Nuclei were stained with 4',6-diamidino-2-phenylindole (DAPI) which was purchased from Molecular Probes (Life Technologies, Paisley, UK). Seedlings were vacuum infiltrated with 1.0µM DAPI according to the manufacturer’s instructions. For DAPI fluorescence the excitation wavelength was 364 nm and the emission filters were set to the range 410 to 490 nm.

REFERENCE LIST

1. Cecchini E, Gong ZH, Geri C, Covey SN, Milner JJ (1997) Transgenic Arabidopsis lines expressing gene VI from cauliflower mosaic virus variants exhibit a range of symptom-like phenotypes and accumulate inclusion bodies. Mol Plant-Microbe Interact 10: 1094-1101.

2. Geri C, Love AJ, Cecchini E, Barrett SJ, Laird J et al. (2004) Arabidopsis mutants that suppress the phenotype induced by transgene-mediated expression of cauliflower mosaic virus (CaMV) gene VI are less susceptible to CaMV- infection and show reduced ethylene sensitivity. Plant Mol Biol 56: 111-124.

3. Mou Z, Fan WH, Dong XN (2003) Inducers of plant systemic acquired resistance regulate NPR1 function through redox changes. Cell 113: 935-944.

4. Bazzini AA, Mongelli VC, Hopp HE, del Vas M, Asurmendi S (2007) A practical approach to the understanding and teaching of RNA silencing in plants. Electronic J Biotechnology 10: 178-190.

5. Nakagawa T, Kurose T, Hino T, Tanaka K, Kawamukai M, et al. (2007) Development of series of gateway binary vectors, pGWBs, for realizing efficient construction of fusion genes for plant transformation. J Bioscience Bioengineering 104: 34-41.

6. Cecchini E, Al Kaff NS, Bannister A, Giannakou ME, McCallum DG et al. (1998) Pathogenic interactions between variants of cauliflower mosaic virus and *Arabidopsis thaliana*. J Exp Bot 49: 731-737.

7. Grant JJ, Yun BW, Loake GJ (2000) Oxidative burst and cognate redox signalling reported by luciferase imaging: identification of a signal network that functions independently of ethylene, SA and Me-JA but is dependent on MAPKK activity. Plant J 24: 569-582.

8. Truman W, de Zabala MT, Grant M (2006) Type III effectors orchestrate a complex interplay between transcriptional networks to modify basal defence responses during pathogenesis and resistance. Plant J 46: 14-33.

9. Love AJ, Yun BW, Laval V, Loake GJ, Milner JJ (2005) *Cauliflower mosaic virus*, a compatible pathogen of Arabidopsis, engages three distinct defence signalling pathways and activates rapid systemic generation of reactive oxygen species. Plant Physiol 139: 935-948.

10. Glazebrook J, Weigel D (2002) Arabidopsis: A laboratory manual. In: Glazebrook J, Weigel D, editors. Cold Spring Harbor NY: Cold Spring Harbor Press. pp. 86.

11. Grant JJ, Chini A, Basu D, Loake GJ (2003) Targeted activation tagging of the Arabidopsis NBS-LRR gene, ADR1, conveys resistance to virulent pathogens. Mol Plant-Microbe Interact 16: 669-680.

12. Love AJ, Laval V, Geri C, Laird J, Tomos AD et al. (2007) Components of *Arabidopsis* defense- and ethylene-signaling pathways regulate susceptibility to *Cauliflower mosaic virus* by restricting long-distance movement. Mol Plant-Microbe Interact 20: 559-570.

13. Bustin SA, Beaulieu JF, Huggett J, Jaggi R, Kibenge FSB et al. (2010) MIQE precis: Practical implementation of minimum standard guidelines for fluorescence-based quantitative real-time PCR experiments. BMC Mol Biol 11: 74.

14. Dean JD, Goodwin PH, Hsiang T (2005) Induction of glutathione S-transferase genes of *Nicotiana benthamiana* following infection by *Colletotrichum destructivum* and *C. orbiculare* and involvement of one in resistance. J Exp Bot 56: 1525-1533.

15. Yu WC, Murfett J, Schoelz JE (2003) Differential induction of symptoms in Arabidopsis by P6 of Cauliflower mosaic virus. Mol Plant Microbe Interact 16: 35-42.

16. Spoel SH, Mou ZL, Tada Y, Spivey NW, Genschik P et al. (2009) Proteasome-Mediated Turnover of the Transcription Coactivator NPR1 Plays Dual Roles in Regulating Plant Immunity. Cell 137: 860-872.
